# Visual supercompensation following short-term exhaustive exercise 

**DOI:** 10.3389/fphys.2025.1583286

**Published:** 2025-10-22

**Authors:** Ina Shaw, Gerrit Breukelman, Lourens Millard, Musa Lewis Mathunjwa, Phindile Zifikile Shangase, Takalani Clearance Muluvhu, Razieh Khanmohammadi, Gregory A. Brown, Brandon S. Shaw

**Affiliations:** ^1^ School of Sport, Rehabilitation and Exercise Sciences, University of Essex, Colchester, United Kingdom; ^2^ Division of Public Health, University of the Free State, Bloemfontein, South Africa; ^3^ Department of Human Movement Science, University of Zululand, KwaDlangezwa, South Africa; ^4^ Department of Sport, Rehabilitation and Dental Sciences, Tshwane University of Technology, Pretoria, South Africa; ^5^ Department of Motor Behavior and Sport Management, Faculty of Sport Sciences, Urmia University, Urmia, Iran; ^6^ Physical Activity and Wellness Laboratory, Department of Kinesiology and Sports Science, University of Nebraska Kearney, Kearney, NE, United States

**Keywords:** hand-eye coordination, peripheral vision, psychomotor performance, reaction time, visual perception

## Abstract

**Introduction:**

The human visual system plays a critical role in high-performance tasks, including sports and activities requiring visuomotor performance. While supercompensation is well-documented in aerobic exercise, its effects on visual performance following anaerobic exercise remain unclear. This study investigates whether short-term exhaustive exercise enhances post-exercise visual performance, with a focus on accommodation facility, saccadic eye movements, speed of recognition, peripheral awareness, hand-eye coordination, and visual memory.

**Methods:**

A randomised controlled trial was conducted with 128 participants. Participants completed baseline visual assessments followed by a 30-s Wingate Anaerobic Test (n = 68) or no intervention (control group) (n = 60). The same visual tests were repeated immediately post-exercise. Statistical analyses included t-tests, analysis of variance (ANOVA), and effect size calculations (Cohen’s d). Multiple comparison corrections (Bonferroni and Holm) were applied to control for family-wise error rates.

**Results:**

The experimental group demonstrated significantly greater improvements in visual performance compared to the control group (P < 0.05). Large effect sizes were observed for hand-eye coordination (ES = 1.539), accommodation facility (ES = 1.138), speed of recognition (ES = 1.007), and peripheral awareness (ES = 0.823). Moderate effect sizes were noted for saccadic eye movements for both the left and right charts (ES = 0.679). Post-hoc multiple comparison corrections confirmed significant improvements in speed of recognition, hand-eye coordination, and peripheral awareness (P < 0.000), while accommodation facility showed marginal significance before correction but became non-significant afterward. Visual memory did not significantly differ between groups (P = 0.065).

**Conclusion:**

This study highlights that short-term exhaustive exercise can induce transient enhancements in visual performance, particularly in tasks requiring rapid recognition, coordination, and peripheral awareness. Whether similar effects occur in athletes, who likely start from a higher baseline of performance than physically inactive individuals participants, remains unknown. Such visual supercompensation could be valuable for athletes and professionals in fast-paced environments, offering an opportunity to optimise visual-motor function before performance-critical tasks.

## 1 Introduction

The human visual system is essential for everyday activities (West et al., 2002; Wood et al., 2011), particularly in sports and high-performance tasks that demand rapid visual processing, and motor responses (Miller and Clapp, 2011; Millard et al., 2020a; Stine et al., 1982). Visual performance encompasses various parameters, including speed of recognition, peripheral awareness, hand-eye coordination and accommodation facility. For example, an athlete, such as a football/soccer player, can enhance accommodation ability, which is the rapid adjustment of focus between near and far objects, through targeted vision training (e.g., alternating focus between close and distant targets using Hart Charts). This improves their ability to quickly shift focus from a nearby teammate to a distant opponent, enhancing on-field decision-making, and reaction speed (Zwierko et al., 2014). Enhancing these abilities through training, or physiological interventions has become an area of growing interest in sports science, and neurophysiology (Lochhead et al., 2024).

Recent studies have examined the effects of acute exercise on visual performance, yielding intriguing results. While recent studies have explored acute exercise effects on visual performance in athletes, demonstrating enhancements in visuomotor coordination,and reaction time (Hülsdünker et al., 2021; Zwierko et al., 2014), there remains a critical gap in understanding whether similar visuomotor supercompensation occurs in physically inactive individuals, who lack the baseline adaptations of trained populations. This study addresses this gap by investigating the impact of a short-term exhaustive anaerobic exercise bout on visuomotor performance in physically inactive adults, focusing on accommodation facility, saccadic eye movements, speed of recognition, peripheral awareness, hand-eye coordination, and visual memory. However, it is important to distinguish between physically inactive individuals, recreationally active individuals, or athletes, as the latter two may have baseline visual performance and physiological responses to exercise that differ markedly from those of physically inactive individuals. Athletic training is known to enhance visual-motor coordination, peripheral awareness, and reaction time due to repeated exposure to high-speed, visually demanding tasks and neuromuscular adaptations (Clark et al., 2020; Zwierko et al., 2014). In contrast, physically inactive individuals may exhibit distinct adaptive responses to acute exercise, potentially attributable to lower baseline activation of visual-cognitive pathways (Bullock et al., 2017). Therefore, investigating visual performance supercompensation in physically inactive individuals is essential to determine whether short-term exhaustive exercise can enhance visual capabilities in this population or elicit different patterns of response compared to trained individuals. Studying physically inactive individuals first allows for a clearer understanding of the fundamental effects of short-term exhaustive exercise on visual performance, without the confounding influence of prior athletic training, which may independently enhance visual-motor abilities (Shaw et al., 2024). Within physically inactive populations, as utilised in this study, Shaw et al. (2022) previously found that prolonged maximal running significantly improved visual functions, including accommodation facility, saccadic eye movements, and peripheral awareness. Similarly, Shaw et al. (2024) reported improvements in visual tasks following maximal aerobic exercise in females, except for visual memory. These findings suggest that intense exercise may enhance visual-motor abilities, possibly due to increased cortical excitability (Shaw et al., 2022). Beyond exercise, Hülsdünker et al. (2021) demonstrated that stroboscopic training could improve visuomotor reaction times in elite youth athletes, primarily by accelerating visual perception and processing rather than affecting motor execution.

Supercompensation is a well-documented phenomenon in sports science, describing the temporary enhancement of physiological functions following a period of intense exertion, and recovery (Cardoos, 2015; Matsui et al., 2011). While this principle has been widely applied to aerobic exercise, its effects on cognitive and sensory systems, particularly vision, are not well understood. Some studies suggest that acute physical exertion may temporarily enhance cognitive and sensory processing by increasing cerebral blood flow, neuromuscular activation, and neurotransmitter release, which could extend to improvements in recognition speed, coordination, and other key visual functions (Chang et al., 2016). The early work of Russian stress and exercise physiologists, described “supercompensation” as the process by which the body recovers from exercise-induced fatigue and adapts by improving performance capacity beyond its previous baseline (Viru, 1985). Thus, the term “visual supercompensation” in this context refers to the temporary enhancement of visual performance following a period of intense physical exertion. Drawing from the well-established supercompensation model in exercise physiology, where the body rebounds to a higher level of function after a training stimulus and recovery, visual supercompensation suggests that certain visual and visuomotor functions (e.g., reaction time, hand-eye coordination, peripheral awareness) may also temporarily improve due to acute neuromuscular and neurocognitive activation induced by exhaustive exercise.

This is because previous supercompensation studies have shown that improvements in physiological mechanisms may directly impact improvements in vision. Notably, exhaustive exercise has been shown to induce glycogen supercompensation not only in skeletal muscle but also in brain regions associated with motor control and cognitive processing (Matsui et al., 2011), potentially facilitating enhanced visual-cognitive function. Furthermore, glycogen-depleting exercise followed by carbohydrate intake has been shown to promote glycogen supercompensation in skeletal muscle via sustained activation of glycogen synthase and AMP-activated protein kinase (AMPK) (Hingst et al., 2018), a mechanism that may also support enhanced neuromuscular and visual performance. Short-term high-intensity exercise training has also been shown to enhance protein stability and reduce cellular stress markers, thereby promoting a favourable adaptive environment for neurophysiological function (Hinkley et al., 2017), which may contribute to improvements in visual processing.

Previous research has also explored the relationship between physical exertion and cognitive performance, demonstrating that exercise can acutely enhance reaction time, attention, and decision-making capabilities. Further, exercise intensity appears to differentially affect perception and decision tasks, with increased workload improving decision task performance but reducing perception task performance (Paas and Adam, 1991). In this regard, high-intensity aerobic exercise has been shown to improve perceptual discrimination and non-decisional processes while influencing decision strategy (Davranche et al., 2024). Acute intense exercise significantly decreases reaction time, suggesting enhanced cognitive-motor connectivity (Roach et al., 2014). Recent studies support the positive effects of acute aerobic exercise on cognitive function in older adults. A single session of moderate-intensity aerobic exercise can improve mnemonic discrimination performance, suggesting benefits for hippocampal-specific memory function in healthy older adults (Callow et al., 2023). Acute aerobic exercise also enhances selective attention by reducing neural processing of unattended stimuli and increasing processing of attended stimuli (Ligeza et al., 2023). Both aerobic and resistance exercises performed at low to moderate intensities have been shown to improve memory in cognitively healthy elderly individuals (Griebler et al., 2022). However, the duration of exercise may impact cognitive benefits differently. While 15 min of aerobic exercise can induce beneficial changes in self-perceived arousal, exercise exceeding 15 min may negatively impact visual recognition memory accuracy (Hacker et al., 2020). These studies collectively demonstrate that acute exercise can enhance various cognitive functions across different age groups, supporting its potential benefits for activities requiring quick decision-making and reactions.

However, findings remain mixed, with some studies reporting transient impairments in vision due to fatigue, while others highlight performance benefits resulting from increased arousal and neurophysiological activation (Lambourne and Tomporowski, 2010). This study offers a novel perspective on the interaction between physiological fatigue, and particularly short-term exhaustive exercise, and sensory performance. Insights into the effects of acute exhaustive exercise on visual function may hold substantial relevance for athletes, military personnel, and individuals in high-stakes professions where optimal visuospatial performance is essential for safety and efficacy. Nonetheless, the extent to which these findings generalize to athletic populations, who typically demonstrate superior baseline visuospatial capabilities compared to physically inactive individuals, is uncertain and warrants further investigation in future research. Thus, this study aims to examine the effects of anaerobic exercise on post-exercise visual performance supercompensation. By evaluating changes these changes, we aim to determine whether forms of physical exertion beyond aerobic exercise can temporarily improve visual abilities. We hypothesise that acute exercise will lead to measurable improvements in visual performance metrics in physically inactive individuals. Specifically, we expect that multiple measures of visual performance, such as accommodation facility, saccadic eye movement, speed of recognition, peripheral awareness, hand-eye coordination, and visual memory will show enhancement following a period of anaerobic exercise compared to baseline. These hypotheses align with previous findings suggesting that physical activity can positively influence visual function.

## 2 Materials and methods

### 2.1 Study subjects and study design

**FIGURE 1:**
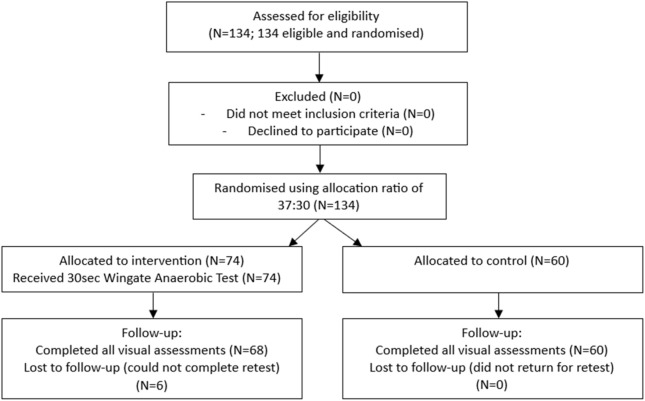
CONSORT flow chart for study on visual supercompensation following short-term exhaustive exercise.

This randomized controlled trial adhered to the Consolidated Standards of Reporting Trials (CONSORT) 2010 guidelines to ensure transparent reporting. A CONSORT-style flow chart (Figure 1) outlines the participant flow, including recruitment, randomization, follow-up, and analysis. Initially, 134 participants were assessed for eligibility, and were randomised using random allocation software into an experimental group (N = 68) receiving a 30-s Wingate Anaerobic Test or a non-intervention control group (n = 60). Due to the nature of the exercise intervention, the study initially recruited more participants for the short-term exhaustive exercise experimental group (N = 37 males, N = 37 females).

The sample size was determined using G*Power (version 3.1.9.7, Universität Düsseldorf, Germany) to detect a large effect size (Cohen’s d = 0.8) at α = 0.05 with 80% power, requiring 28 participants per group (56 total) for a single outcome without correction for multiple comparisons. For six visual performance outcomes, a Bonferroni-corrected α = 0.0083 necessitated 43 participants per group (86 total), including a 10% dropout adjustment. The final sample of 128 participants (61 males, 67 females) exceeded these estimates, ensuring robust power to detect significant visual performance improvements in a physically inactive population, accommodating potential attrition, and supporting secondary analyses like Principal Component Analysis. This sample size aligns with prior studies (Ando et al., 2008; Shaw et al., 2022; Shaw et al., 2024), enhancing the study’s ability to detect meaningful effects while maintaining generalisability within the physically inactive cohort.

This study included both males and females to ensure a comprehensive understanding of how short-term exhaustive exercise influences post-exercise visual performance supercompensation across all sexes. The inclusion criteria required participants to have a minimum of 20/20 vision, either unaided or corrected with soft contact lenses during the experiment. Additionally, participants must have had no prior experience with sports vision testing (Millard et al., 2020b), and must not have engaged in any structured sport or exercise regimen in the past 6 months (i.e., physically inactive (Tremblay et al., 2017)), as defined as less than 150 min of moderate-intensity or 75 min of vigorous-intensity physical activity per week, per WHO guidelines (Bull et al., 2020). Participants were specifically selected from a physically inactive population to control for the potential confounding effects of physical activity on visual performance. Previous research suggests that regular engagement in sports or physical exercise can enhance certain aspects of visual function (Ha et al., 2015). To isolate the effects under investigation and avoid bias introduced by varying activity levels, physically inactive individuals were chosen. This approach ensures that observed outcomes are not influenced by prior physical training, or sport-related visual enhancements, allowing for clearer interpretation of the study results.

Exclusion criteria included the absence of 20/20 vision (either unaided or corrected with soft contact lenses), any visual disease or infection, physical disability, or psychosocial distress (e.g., depression, anxiety, severe stress, adjustment disorders, or other mental illnesses under active treatment) (West et al., 2002). In this regard, prior to participation in the study, participants underwent an optometric assessment to ensure 20/20 vision. Depth perception and visual acuity were measured using Spectrum Eyecare software (Version 6.0.0, Digital Optometry, South Africa). Participants were also excluded if they had any absolute or relative contraindications to exercise or testing (Riebe et al., 2018). Before participation, all individuals received both written and oral information regarding the study’s objectives, data collection, and data management. Informed written consent was obtained, and participants were allowed to withdraw from the study at any time.

### 2.2 Test procedure

For descriptive purposes, participants were evaluated for height, body weight, and body mass index (BMI) as per the International Society for the Advancement of Kinanthropometry (ISAK) guidelines (Norton and Olds, 1996). Standing height was measured to the nearest 0.1 cm (cm) using a portable stadiometer (Holtain Limited, Crymych, Dyfed, United Kingdom) with participants standing barefoot. Body weight was measured to the nearest 0.5 kg (kg) using a portable digital scale (Dismed, Indianapolis, IN, United States). Participants were barefoot and wore minimal clothing during the measurement. Body mass index (BMI) was calculated as weight in divided by height in meters squared (kg·m^-2^), providing an assessment of weight relative to height.

### 2.3 Outcome measures

The visual performance tasks employed in this study assess visuomotor integration, a process where visual processing (e.g., accommodation speed, saccadic accuracy, peripheral detection) directly informs motor outputs as is standard in applied sports vision research (Millard et al., 2020a). The use of a physically inactive population, with presumed lower baseline motor proficiency, and a control group further controls for non-specific motor improvements or learning effects. While pure isolation of visual processing would require advanced neuroimaging, the current design prioritises ecological validity in assessing visually guided performance relevant to real-world tasks.

#### 2.3.1 Accommodation facility

This study conducted at the Visio Spatial Intelligence (VSI) Laboratory at the University of Zululand, South Africa, employed the Hart Near-Far Rock Test to evaluate accommodation facility, which refers to the eye’s ability to adjust its refractive power for clear vision at varying distances (Mcbrien and Millodor, 1987). The large Hart Chart was positioned at head height, 3 m away from participants (Du Randt et al., 2016). Each participant held a smaller chart at arm’s length and was instructed to alternate reading the first letter of the first line from the far and near charts. This process continued for 30 s, after which errors were subtracted from the score to determine the result. An error was defined as an incorrect letter identification (Du Randt et al., 2016). This assessment has been previously shown to exhibit a reliability coefficient of 0.724 (Millard et al., 2024), and 0.723 (Bonnesse, 2016).

#### 2.3.2 Saccadic eye movement

In this assessment, no eye tracker or electronic tracking device was used. Instead, saccadic eye movements, characterised by rapid, ballistic shifts in fixation, were evaluated using a standardised, chart-based Adapted Saccadic Chart Test protocol adapted from established visual skills assessments (Purves et al., 2001). Two charts were placed 1 m apart on a board, 3 m from the participants. Participants were instructed to read the first letter on the far-left chart, shift their gaze rapidly to the far-right chart, and continue this process for 30 s. Saccadic effectiveness was measured by the number of correct gaze shifts, minus errors, reflecting saccadic precision and speed, consistent with established protocols (Zwierko et al., 2014). Saccadic eye movements were recorded manually through participant verbal reporting of fixated letters, with the assessor counting correct shifts minus errors. Errors were deducted from the final score with an error is defined as an incorrect identification or failure to accurately shift gaze between targets. Errors indicate issues with saccadic accuracy, such as overshooting or undershooting, rather than motor response. To prevent memorisation, standardised adjustable saccadic eye movement charts with vertically arranged letters were used (Du Randt et al., 2016). This assessment demonstrates a reliability coefficient of 0.703 (Bonnesse, 2016).

#### 2.3.3 Speed of recognition

Speed of recognition was measured using the Batak Pro Evasion Program (Lobier et al., 2013; Quotronics Limited, 2011). The programme randomly illuminated 12 LED lights for one second, and participants were required to strike the lights while they remained lit. If a light flickered instead of staying illuminated, participants were not to strike it; doing so resulted in a five-point deduction (Quotronics Limited, 2011). Additionally, when all centre lights flickered, participants had to evade the small central infrared beam. Failure to do so resulted in another five-point deduction. The Batak Pro microcomputer automatically calculated the final score as the total number of correct hits out of 100 on the Batak Pro Evasion Program. The score reflects visual processing speed and accuracy, not direct reaction time, consistent with established methods (Lobier et al., 2013). This evaluative procedure exhibits a reliability coefficient of 0.946 (Bonnesse, 2016).

#### 2.3.4 Hand-eye coordination

Hand-eye coordination was evaluated using the Ball Wall Toss Test (Rizzo et al., 2019) A mark was placed 2 m from a wall where participants were required to throw and catch a standard tennis ball while alternating hands for 30 s (Du Randt et al., 2016). Although the tennis ball task includes reaction time, and motor components, it primarily evaluates hand-eye coordination through the detection, and interception of stimuli in the peripheral visual field, with performance metrics centred on successful catches rather than pure motor speed, aligning with validated sports vision protocols (Kruger et al., 2009; Millard et al., 2020a). The number of successful catches was recorded. This evaluative procedure exhibits a reliability coefficient of 0.708 (Bonnesse, 2016).

#### 2.3.5 Peripheral awareness

Peripheral awareness was measured using the Accumulator Program on the Batak Pro (Kruger et al., 2009; Lobier et al., 2013). In this test, random LED targets illuminated and remained lit until struck by the participant. After being struck, another LED target immediately illuminated for a period of 60 s. The Batak Pro microcomputer automatically calculated the final score at the end of the test (Quotronics Limited, 2011). This measurement protocol demonstrates a reliability coefficient of 0.885 (Bonnesse, 2016).

#### 2.3.6 Visual memory

Visual memory was assessed using the Flash Memory Program on the Batak Pro (Quotronics Limited, 2011; Shurgin, 2018). In this test, six targets lit up for half a second, after which participants were required to recall the illuminated targets and the order in which they appeared (Quotronics Limited, 2011; Millard et al., 2021). The Batak Pro microcomputer recorded the maximum score at the end of the test. The reliability of this test was previously established at 0.735 (Bonnesse, 2016).

### 2.4 Interventions

One week after the baseline visual task assessments, participants completed a short-term exhaustive exercise (i.e., a 30-s Wingate Anaerobic event), followed immediately by the same test battery described above. The short-term exhaustive exercise was conducted on a cycle ergometer (Model 834E, Monark Exercise AB, Vansbro, Sweden) following the protocol by Inbar et al. (1996). Participants performed a 5-min warm-up at the ergometer’s inertial resistance, with two 4-s sprints in the final seconds of the second and fourth minutes. After a 10-min rest, participants pedalled “all-out” for 30 s against a resistance of 0.09 kg kg^-1^ body mass, with verbal encouragement provided throughout (Inbar et al., 1996). Power output was recorded every second. The highest external power output within the first 5 seconds of the test was considered peak power (PP), while the mean power (MP) was calculated as the average power over the 30-s test (Inbar et al., 1996). The fatigue index (FI) was determined as the percentage decline from PP to the lowest power recorded [(PP - lowest power)/PP × 100] (Inbar et al., 1996).

Immediately after completing the short-term exhaustive exercise, participants resumed the sports vision test battery. Control group participants completed the same test battery without prior treatment to assess the potential for a learning effect.

### 2.5 Statistical analyses

#### 2.5.1 Within-group analysis

All statistical analyses were conducted using IBM SPSS Statistics (Version 27, NY, United States). All measures with p-values ≤0.05 were considered statistically significant. Descriptive statistics (mean ± standard deviation) were calculated for all outcome variables. To evaluate the effect of acute exercise on visual performance within-groups, paired t-tests to compare pre-, and post-intervention scores within each group. Effect sizes (Cohen’s d) were also calculated and the standardised effect sizes were classified as small (<0.20), moderate (0.20–0.79), and large (≥0.80).

#### 2.5.2 Between-group analysis

To compare the experimental and control groups at baseline, a Welch’s t-test was performed, while independent t-tests were used to compare the post-test scores between the exercise, and control groups. To determine distributions across the metrics and groups, a Shapiro-Wilk normality test was conducted, where P > 0.05 indicated normality, and skewness (<0.5 indicating approximate symmetry, >0.5 indicating right skew, and < −0.5 indicating left skew). In addition an Analysis of Variance (ANOVA) was used with Time (pre-vs. post-intervention) as the within-subjects factor and Group (exercise vs. control) as the between-subjects factor. Separate ANOVAs were conducted for each dependent variable, including accommodation, saccadic eye movements, speed of recognition, hand–eye coordination, peripheral awareness, and visual memory. Multiple comparison corrections, including Bonferroni and Holm methods, were applied to control for family-wise error rates. Both Bonferroni and Holm corrections were applied to multiple comparisons to provide complementary perspectives, as Bonferroni offers a more conservative control of Type I error, while Holm maintains strong error control with greater statistical power. Reporting both methods allowed for transparency, and demonstrates the robustness of the key findings across different correction strategies. A Principal Component Analysis (PCA) was included to reduce the dimensionality of the visual performance data. This approach allowed us to identify underlying patterns across multiple correlated visual measures. By extracting key components, we were able to create composite scores representing overall visual performance. This supports our hypothesis by assessing whether exercise produces global improvements rather than isolated effects.

## 3 Results

### 3.1 Participant demographics

The short-term exhaustive exercise experimental group (n = 68) had a mean age of 23.78 ± 2.78 years and consisted of 37 females and 31 males. The non-intervention control group (n = 60) had a mean age of 25.8 ± 4.53 years, with an equal distribution of 30 females and 30 males. Table 1 presents the participant demographic data. Due to the nature of the exercise intervention, the study initially recruited 20% more participants for the short-term exhaustive exercise experimental group (n = 37 males, n = 37 females). All female participants returned for the retest, while six male participants did not. All the non-intervention control group participants returned for the retest.

**TABLE 1:** Participant (n = 128) demographics.

Age (years)	23.76 ± 2.80
Height (cm)	171 ± 16
Body weight (kg)	69.04 ± 14.54
Body mass index (BMI) (kg/m^2^)	23.34 ± 4.16

Data reported as means ± standard deviations (SD). cm: centimetres; kg: kilogrammes; kg/m^2^: kilogrammes per square metre.

### 3.2 Within-group analyses

For both the experimental, and control groups, baseline data exhibited a mix of normal, and non-normal distributions. For the control group, the Shapiro–Wilk test indicated that accommodation facility (W = 0.08, P = 0.370), and visual memory (W = 0.191, P = 0.097) did not significantly deviate from normality, and were therefore considered normally distributed. However, saccadic eye movement (W = 0.381, P = 0.014), speed of recognition (W = 0.536, P = 0.002), peripheral awareness (W = −2.520, P < 0.001), and hand-eye coordination (W = 0.111, P = 0.020) significantly deviated from normality, and were therefore not normally distributed. Regarding the experimental group, peripheral awareness (W = −0.402, P = 0.261), did not significantly deviate from normality. However, accommodation facility (W = 0.767, P = 0.002), saccadic eye movement, (W = 0.407, P = 0.017), speed of recognition (W = 0.593, P < 0.001), hand-eye coordination (W = −0.060, P = 0.025), and visual memory (W = −0.912, P = 0.001) significantly deviated from normality.

Within-groups, the paired-samples t-tests demonstrated that speed of recognition, hand-eye coordination, and peripheral awareness remained highly significant (P < 0.000) in the experimental group, while visual memory showed only a small, non-significant effect (ES = 0.214, P = 0.063) (Table 2). The effect size findings in Table 2 highlight the substantial impact of short-term exhaustive exercise on visual performance supercompensation. In the experimental group, large effect sizes were observed for hand-eye coordination (ES = 1.539), accommodation facility for both the large and small charts (ES = 1.138), speed of recognition (ES = 1.007), and peripheral awareness (ES = 0.823) indicating strong improvements in these areas post-exercise. Moderate effect sizes were observed for saccadic eye movement for both the left and right charts (ES = 0.679), suggesting notable enhancements in these visual functions. In contrast, the control group exhibited smaller effect sizes across all measures, with the largest observed in accommodation facility (ES = 0.503) and peripheral awareness (ES = 0.349).

**TABLE 2:** Effects of short-term exhaustive exercise on post-exercise visual performance supercompensation.

Variables	Short-term exhaustive exercise experimental group (n = 68)				Non-intervention control group (n = 60)				Between-groups (mean change)
	Pre-test	Post-test	Within-groups p-value	ES	Pre-test	Post-test	Within-groups p-value	ES	p-value
Accommodation Facility - Large Chart	15.56 ±2.53	18.75 ±3.05	0.000^a^	1.138	35.80 ±4.53	38.00 ±4.21	0.000^a^	0.503	0.036^a^
Accommodation Facility - Small Chart	15.56 ±2.53	18.75 ±3.05	0.000^a^	1.138	35.80 ±4.53	38.00 ±4.21	0.000^a^	0.503	0.036^a^
Saccadic Eye Movement - Left Chart	20.57 ±4.34	23.59 ±4.54	0.000^a^	0.679	38.20 ±6.86	40.63 ±7.39	0.001^a^	0.341	0.435
Saccadic Eye Movement - Right Chart	20.57 ±4.34	23.59 ±4.54	0.000^a^	0.679	38.20 ±6.86	40.63 ±7.39	0.001^a^	0.341	0.435
Speed of Recognition	21.76 ±16.82	41.46 ±21.95	0.000^a^	1.007	28.80 ±18.4	30.37 ±19.89	0.066	0.082	0.000^a^
Peripheral Awareness	60.21 ±5.99	69.85 ±6.54	0.000^a^	0.823	65.37 ±11.55	68.80 ±7.73	0.004^a^	0.349	0.000^a^
Hand-Eye Coordination	20.47 ±4.74	24.28 ±4.51	0.000^a^	1.539	22.97 ±5.29	23.63 ±5.70	0.226	0.121	0.000^a^
Visual Memory	39.90 ±6.71	41.31 ±6.38	0.063	0.214	41.57 ±5.05	43.40 ±6.05	0.001^a^	0.329	0.065

Data reported as means±standard deviations (SD).

^a^ Statistical significance was set at *P* ≤ 0.05; ES: effect size, where <0.20 is considered small, 0.20–0.79 moderate and ≥0.80 large.

### 3.3 Between-group analyses

Baseline comparisons using independent t-tests showed significant differences between intervention and control groups for accommodation facility (P < 0.001), saccadic eye movements (P < 0.001), speed of recognition (P = 0.026), hand–eye coordination (P = 0.006), and peripheral awareness (P = 0.003), but not visual memory (P = 0.115).

Between groups, the independent-samples t-tests showed that the anaerobic group demonstrated significantly (P < 0.05) greater improvements across most visual performance measures compared to the control group. Statistical comparisons between groups indicated significant differences in accommodation facility, speed of recognition, hand-eye coordination and peripheral awareness (Table 2). ANOVA test results demonstrated that there was a significant difference between the mean difference scores of accommodation facility (F (1,128) = 4.514; P = 0.036; 95% CI: 0.04–1.94), speed of recognition (F (1,128) = 83.52; P = 0.000; 95% CI: 14.36–21.89), peripheral awareness (F (1,128) = 25.41; P = 0.000; 95% CI: 3.68–8.74), and hand-eye coordination (F (1,128) = 24.30; P = 0.000; 95% CI: 1.85–4.43), but not saccadic eye movement (F (1,128) = 0.61; P = 0.435; 95% CI: 0.95–2.11), and visual memory (F (1,128) = 0.20; P = 0.654; 95% CI: 2.23–1.39).

After applying *post hoc* multiple comparison corrections analysis using both Bonferroni and Holm corrections, speed of recognition, hand-eye coordination and peripheral awareness show significant differences (P = 0.000) even after both Bonferroni and Holm corrections, accommodation facility show marginal significance in the original analysis (P = 0.042) but become non-significant after corrections (Bonferroni: P = 0.336 and Holm: P = 0.210). Both saccadic eye movement, and visual memory show no significant differences between groups after corrections (P = 1.000, respectively).

To support our hypothesis by assessing whether exercise produces global improvements rather than isolated effects, the PCA demonstrated the variance ratio for each principal component, determining the optimal number of components to retain in the PCA. Specifically for this study, PC1 explained 33.66% of variance and was loaded by accommodation facility, and saccadic variables. PC2 explained 21.28% of variance, and was dominated by speed of recognition variables, while PC3 explained 13.84% of variance, and was primarily driven by hand/eye coordination variables. In this regard, four components (i.e., accommodation facility, saccadic eye movement, speed of recognition, and peripheral awareness) were needed to explain 80.35% of the total variance, and demonstrated that exercise produces global improvements in visual performance rather than isolated effects.

## 4 Discussion

This study examined the effects of short-term exhaustive exercise on post-exercise visual performance supercompensation, with a specific focus on accommodation facility, saccadic eye movements, speed of recognition, peripheral awareness, hand-eye coordination and visual memory. The findings provide compelling evidence that anaerobic exercise can significantly enhance key aspects of visual performance, supporting the hypothesis that physiological supercompensation extends beyond traditional strength and aerobic adaptations.

The results demonstrated significant improvements in several visual parameters following short-term exhaustive exercise. Specifically, hand-eye coordination, accommodation facility, speed of recognition, and peripheral awareness exhibiting large effect sizes, suggesting a strong link between anaerobic exertion and enhanced visual-motor performance. Peripheral awareness and saccadic eye movements also improved significantly, albeit with moderate effect sizes. However, visual memory did not show significant improvement, suggesting that the cognitive demands of memory retention may not be as directly influenced by acute physical exertion as other visual-motor skills.

While the concept that acute exhaustive exercise may enhance visual performance supercompensation is novel, recent studies have provided evidence of significant improvements in visual performance following aerobic exercise. Shaw et al. (2024) observed improvements in multiple visual tasks after maximal aerobic exercise, although only visual memory remained unaffected. Another study by Shaw et al. (2022) reported enhanced visual performance following a prolonged maximal bout of running. These findings suggest that aerobic exercise can positively impact visual-motor abilities, potentially due to increased cortical excitability or improved neural processing. Additionally, Matsui et al. (2011) observed glycogen supercompensation in the brain following exhaustive exercise, which may contribute to enhanced cognitive function. However, our study extends these findings by demonstrating that anaerobic exercise, rather than aerobic exercise alone, can yield substantial visual performance benefits.

These improvements align with previous research indicating that intense physical activity can acutely elevate neuromuscular activation, neurotransmitter release and cerebral blood flow, which may contribute to enhanced visual function. In this regard, research indicates that intense physical activity can acutely enhance visual function through various mechanisms. Exercise has been shown to increase cortical glutamate and gamma-aminobutyric acid (GABA) levels in the visual cortex, potentially expanding neurotransmitter pools (Maddock et al., 2016). Physical activity combined with cognitive tasks can modulate visual parameters, improving accommodative facility while potentially impairing near stereoacuity and eye-hand coordination (Vera et al., 2017). Recent studies demonstrate that physical activity enhances visual target detection, possibly by reducing interneuronal correlation and improving spatial resolution in early visual processing (Weischner et al., 2024). Additionally, exercise-induced increases in cerebral blood volume may contribute to improved brain function and motor performance (Perrey, 2013). Acute exercise has also been shown to enhance cognitive and sensorimotor processes. Exhaustive running can facilitate adaptation in manual tracking tasks, potentially due to increased cortical efficiency (Mierau et al., 2009). Exercise-induced arousal improves sensory discrimination but may not affect executive processing (Lambourne et al., 2010). Moderate-intensity exercise generally enhances cognitive function, as evidenced by improved response times in both congruent and incongruent Stroop task conditions (Chang et al., 2016). This improvement may be attributed to increased attentional resource allocation and conflict detection processes, reflected in ERP components like P3 and N450 (Chang et al., 2016). The concept of “exercise priming” suggests that positioning aerobic exercise near therapeutic activities could enhance treatment outcomes through neural plasticity-promoting mechanisms (Moriarty et al., 2019). These findings suggest that physical activity can acutely influence neuronal activity and visual perception, although the optimal exercise parameters for enhancing visual function remain to be determined.

It should be noted that several baseline differences between the intervention and control groups were observed, particularly for accommodation facility, saccadic eye movements, speed of recognition, hand-eye coordination, and peripheral awareness. These differences likely reflect natural variability within our physically inactive sample, despite random allocation. While this introduces a potential limitation, our analyses focused on within-group changes and between-group differences in change scores, thereby mitigating the impact of baseline disparities on the interpretation of post-intervention effects. Importantly, the inclusion of corrections for multiple comparisons, and PCAs strengthens confidence that the observed improvements in visual performance were attributable to the intervention rather than pre-existing group differences.

Despite the robust findings, this study has some limitations. The short-term nature of the intervention does not provide insights into the long-term effects of exhaustive exercise on visual performance. Future studies should explore whether repeated exposure to anaerobic exercise leads to sustained improvements or adaptive changes in visual processing. Furthermore, the mechanisms underlying the observed enhancements, such as neurotransmitter activity and neurovascular responses, warrant further investigation through neuroimaging and electrophysiological studies. A further limitation of the current study is the potential influence of motor contributions in tasks requiring visuomotor integration, as the paradigms (e.g., speed of recognition, hand-eye coordination) involve motor responses that could partially reflect general motor speed improvements post-exercise. While the study design minimised this through simple response modalities, and a physically inactive sample to reduce baseline motor proficiency effects, it did not independently assess general motor speed. Future research could incorporate control tasks, such as auditory reaction time tests, to isolate visual processing contributions from motor performance, enhancing the specificity of findings regarding visual supercompensation (Vera et al., 2017). Another limitation is the potential for residual learning effects, as evidenced by the control group’s improvements in most visual performance tasks from pre-to post-measurement, despite a familiarisation session. While statistical analyses accounted for these effects, future studies could employ parallel task versions or extended familiarization to further minimise practice-related confounds, enhancing the interpretability of intervention effects.

## 5 Conclusion

This study provides compelling evidence that short-term exhaustive exercise can significantly enhance multiple aspects of visual performance, notably speed of recognition, hand-eye coordination, and peripheral awareness, in a physically inactive adult population. These enhancements suggest practical utility in contexts where rapid visual-motor processing is critical, such as competitive sports, tactical military operations, aviation, and other high-stakes environments.

Importantly, the observed effects may be harnessed in applied settings as part of warm-up, or priming strategies to temporarily sharpen visual-motor readiness before performance-critical tasks. For instance, integrating brief high-intensity anaerobic efforts before gameplay or mission execution could offer a low-cost, non-invasive method of boosting perceptual efficiency.

While this study was conducted under controlled laboratory conditions with physically inactive participants, the magnitude of observed effects, and their alignment with known neurophysiological mechanisms (e.g., increased cortical excitability, neurovascular activation) support the plausibility of generalisation to broader populations, including athletes and occupational professionals. However, caution should be exercised when extending findings to trained individuals, or complex field settings, as baseline adaptations and environmental variability could moderate outcomes. This is because it is unknown whether similar effects occur in athletes, who likely start from a higher baseline of visual performance.

Future field-based studies are needed to confirm whether these effects persist under ecological conditions, and across diverse populations. Nonetheless, these findings offer foundational evidence for the potential of targeted physical exertion as a tool for transiently enhancing visual performance beyond the lab.

## Data Availability

The raw data supporting the conclusions of this article will be made available by the authors, without undue reservation.
